# Endoscopic ultrasound-guided gallbladder drainage for acute cholecystitis: Long-term outcomes after removal of a self-expandable metal stent

**DOI:** 10.3748/wjg.v23.i4.661

**Published:** 2017-01-28

**Authors:** Ken Kamata, Mamoru Takenaka, Masayuki Kitano, Shunsuke Omoto, Takeshi Miyata, Kosuke Minaga, Kentaro Yamao, Hajime Imai, Toshiharu Sakurai, Tomohiro Watanabe, Naoshi Nishida, Masatoshi Kudo

**Affiliations:** Ken Kamata, Mamoru Takenaka, Masayuki Kitano, Shunsuke Omoto, Takeshi Miyata, Kosuke Minaga, Kentaro Yamao, Hajime Imai, Toshiharu Sakurai, Tomohiro Watanabe, Naoshi Nishida, Masatoshi Kudo, Department of Gastroenterology and Hepatology, Kindai University Faculty of Medicine, Osaka-Sayama 589-8511, Japan

**Keywords:** Endoscopic ultrasound-guided gallbladder drainage, Cholecystitis, Endoscopic ultrasound-guided biliary drainage

## Abstract

**AIM:**

To assess the long-term outcomes of this procedure after removal of self-expandable metal stent (SEMS). The efficacy and safety of endoscopic ultrasound-guided gallbladder drainage (EUS-GBD) with SEMS were also assessed.

**METHODS:**

Between January 2010 and April 2015, 12 patients with acute calculous cholecystitis, who were deemed unsuitable for cholecystectomy, underwent EUS-GBD with a SEMS. EUS-GBD was performed under the guidance of EUS and fluoroscopy, by puncturing the gallbladder with a needle, inserting a guidewire, dilating the puncture hole, and placing a SEMS. The SEMS was removed and/or replaced with a 7-Fr plastic pigtail stent after cholecystitis improved. The technical and clinical success rates, adverse event rate, and recurrence rate were all measured.

**RESULTS:**

The rates of technical success, clinical success, and adverse events were 100%, 100%, and 0%, respectively. After cholecystitis improved, the SEMS was removed without replacement in eight patients, whereas it was replaced with a 7-Fr pigtail stent in four patients. Recurrence was seen in one patient (8.3%) who did not receive a replacement pigtail stent. The median follow-up period after EUS-GBD was 304 d (78-1492).

**CONCLUSION:**

EUS-GBD with a SEMS is a possible alternative treatment for acute cholecystitis. Long-term outcomes after removal of the SEMS were excellent. Removal of the SEMS at 4-wk after SEMS placement and improvement of symptoms might avoid migration of the stent and recurrence of cholecystitis due to food impaction.

**Core tip:** Endoscopic ultrasound-guided gallbladder drainage (EUS-GBD) was recently used to treat acute cholecystitis. The aim of this study was to assess the utility of removal of self-expandable metal stent (SEMS) at 4-wk after EUS-GBD. Twelve patients with acute calculous cholecystitis underwent EUS-GBD with a SEMS. The rates of technical success, clinical success, and adverse events were 100%, 100%, and 0%, respectively. Recurrence was seen in one patient (8.3%). The median follow-up period after EUS-GBD was 304 d. Removal of the SEMS at 4-wk after SEMS placement might avoid migration of the stent and recurrence of cholecystitis due to food impaction.

## INTRODUCTION

Laparoscopic cholecystectomy is the standard treatment for acute cholecystitis caused by cholecystolithiasis[1,2]. For patients at high surgical risk, percutaneous transhepatic gallbladder aspiration (PTGBA) or percutaneous transhepatic gallbladder drainage (PTGBD) can be selected for treatment of cholecystitis. However, the efficacy rate of PTGBA is insufficient (61%-77%), and PTGBD involves an external drainage tube, which decreases the ability of the patient to carry out their normal daily activities[3,4]. Recently, endoscopic ultrasound-guided gallbladder drainage (EUS-GBD) was developed for acute cholecystitis[5-17]. Jang et al[14] showed that EUS-GBD was comparable with PTGBD in terms of its technical feasibility, efficacy, and procedural safety.

The aim of this study was to evaluate the outcomes of EUS-GBD in patients with acute calculous cholecystitis deemed unsuitable for cholecystectomy. The examined procedure used a self-expandable metal stent (SEMS), and we also assessed the long-term outcomes of the procedure following removal of the SEMS.

## MATERIALS AND METHODS

### Patients

Between January 2006 and October 2014, 225 patients with acute cholecystitis due to gallstones visited our hospital. Among these, 101, 18, 32, and 62 patients underwent PTGBA and/or PTGBD, endoscopic naso-gallbladder drainage, emergent surgery and conservative treatment, respectively. The remaining 12 patients with acute calculous cholecystitis, who were deemed unsuitable for cholecystectomy because of poor surgical performance indications and had a risk of self-removal of drainage tube, underwent EUS-GBD. Cases of cholecystitis due to deployment of the metal stent and the cases that cystic duct was obstructed due to advanced cancer were excluded from this study.

The surgical performance indications for all patients were poor (class III or IV on the American Society of Anesthesiologists (ASA) Physical Status classification system). These patients were identified by retrospective review of the medical database of our hospital. Acute calculous cholecystitis was diagnosed in all patients on the basis of the characteristic clinical features (abdominal pain and fever), laboratory data (high level of serum C-reactive protein; CRP), and imaging studies. The study was approved by the institutional review board of the Kinki University Faculty of Medicine, and informed consent was obtained from the patients after explaining to them that we could perform PTGBA, PTGBD, or EUS-GBD.

### EUS-GBD technique

An echoendoscope (GF-UCT240-AL5, Olympus, Tokyo, Japan) was introduced into the stomach or duodenum. The echoendoscope images were used to ensure that gallstones were present in the swollen gallbladder before EUS-GBD was performed. After visualization of the swollen gallbladder adjacent to the antrum or duodenal bulb, the echoendoscope was manipulated until an appropriate puncture route, free from interposing vessels, was identified. The puncture site was selected as the region where the distance between the gastrointestinal tract and the gallbladder was smallest (1 cm or less). When both the stomach and duodenum provided equally good access, the duodenum was selected as the puncture site because it was easier to maintain the scope position at the duodenum than at the stomach.

**Figure 1:**
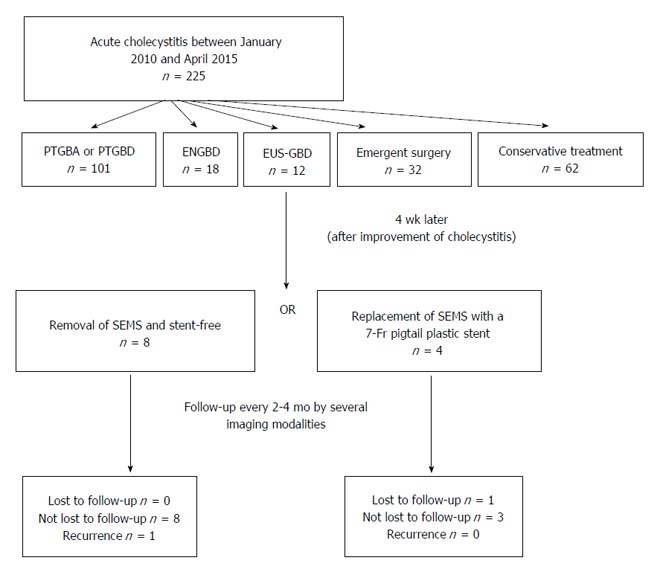
Strategy of endoscopic ultrasound-guided gallbladder drainage procedure. ENGBD: Endoscopic naso-gallbladder drainage; EUS-GBD: Endoscopic ultrasound-guided gallbladder drainage; PTGBA: Percutaneous transhepatic gallbladder aspiration; PTGBD: Percutaneous transhepatic gallbladder drainage; SEMS: Self-expandable metal stent.

The neck or body of the gallbladder was generally chosen as the ideal target, and was then punctured with a 19G needle (EchoTip Ultra, Cook Medical, Limerick, Ireland) under endosonographic guidance. The gallbladder was then irrigated with a saline solution through the 19G needle, using a 20 mL syringe. Irrigation was performed at least ten times, and was continued until the color of the bile became weak. This was performed to prevent peritonitis due to bile leakage immediately after the gallbladder was punctured. Thereafter, a sufficient length of 0.035 inch guidewire (Revowave, Piolax, Kanagawa, Japan) was inserted into the gallbladder lumen until there were more than two coils present. The puncture tract was then serially dilated using either biliary dilation catheters (6F-7F-9F, Soehendra Biliary Dilation Catheter, Cook, Bloomington, IN, United States) or a balloon dilator (Max Pass 4 mm, Olympus, Tokyo, Japan) over the guidewire. If passing dilators or balloons proved difficult, electrocautery was planned to be used. A SEMS (10 mm in diameter, 6 cm in length, Wallflex partially covered stent, Boston Scientific, Natick, MA, United States) was deployed between the gallbladder and the stomach or duodenum. If functional success was obtained, the SEMS was removed and/or replaced with a 7-Fr plastic pigtail stent (4 or 6 cm in length) 4 wk after the original EUS-GBD (Figure 1). Where possible, the stent was replaced after removal of the SEMS in order to keep the fistula considering the possibility of the recurrence. This technique was approved by the institutional review board of the Kinki University Faculty of Medicine.

### Follow-up after EUS-GBD

Several imaging modalities including ultrasonography, computed tomography (CT), fistulography, and/or EUS were performed to determine if gallstones remained in the gallbladder before removal of the SEMS. CT (looking for air images in the gallbladder) and/or fistulography were performed to determine if the fistula remained open 1 wk after removal of the SEMS. After removal of the SEMS, patients were continually followed up by blood tests and imaging modalities every 2-4 mo. It was determined whether the cystic duct was patent before and after removal of the SEMS by performing fistulography and/or EUS.

### Assessment of outcomes

The long-term outcomes of EUS-GBD after removal of SEMS was the primary outcomes in this study. The outcomes assessed were technical and clinical success rates, adverse events rate, and recurrence rate. Technical success was defined as successful stent deployment between the gallbladder lumen and the stomach or duodenum. Clinical success was defined as improvement of typical clinical symptoms within 3 d, with confirmatory laboratory tests, with or without improved radiologic findings[14]. The incidence of the following adverse events was assessed: peritonitis, bile leakage, bleeding, stent migration, and stent occlusion. Recurrence of acute cholecystitis after EUS-GBD was defined on the basis of the characteristic clinical features, laboratory data, and imaging studies.

### Statistical analysis

Continuous variables are expressed as median or mean values with standard deviation or range. All statistical analyses were performed using SAS software version 9.1 (SAS Institute, Cary, NC, United States).

## RESULTS

**Figure 2:**
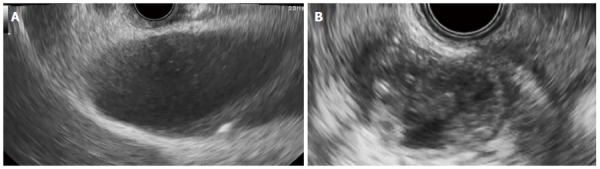
Endosonographic image. A: Endosonographic image before gallbladder drainage. Contents in the gallbladder were mainly sludge without apparent gallstones; B: Endosonographic image at the time of recurrence of cholecystitis. Sludge volume in the gallbladder was more increased than when gallbladder drainage was performed.

Table 1 shows the patients’ characteristics. In total, 12 patients (mean age 76 years, 9 men and 3 women) underwent EUS-GBD. Eight patients were ASA class III, and the others were ASA class IV. One patient had advanced ovarian cancer which expected long-term survival and there was no influence of the tumor on the cystic duct. Blood examination revealed a mean white blood cell (WBC) count of 14525 cells per μL and a mean CRP level of 15.7 mg/dL. All cases were moderate cholecystitis. The diameter of gallstones was less than 10 mm in all patients. The EUS-GBD procedure was performed *via* the stomach or duodenum in three and nine cases, respectively. The distance between the gastrointestinal tract and the gallbladder was 1 cm or less in all cases. Dilation of the puncture site was performed by biliary dilation and/or balloon catheters without using electrocautery. Table 2 shows the outcomes of EUS-GBD. The technical success and clinical success rates were both 100% (12/12), with no adverse events recorded. At day 3 post-EUS-GBD, the mean WBC count and mean CRP were 7075 cells per μL and 2.37 mg/dL, respectively. The SEMS was removed from eight patients 4 wk after the EUS-GBD. In these eight patients, the plastic pigtail stent was not deployed after removal of the SEMS because the guidewire could not be sufficiently inserted due to shrinkage of the gallbladder by the EUS-GBD treatment. In the remaining four patients, the SEMS was replaced with a 7-Fr plastic double pigtail stent 4 wk after EUS-GBD. The median post-EUS-GBD follow-up period for these 12 patients was 304 d. During the follow-up period, one of the patients (8.3%) died due to advanced cancer. At the time the records were subjected to retrospective evaluation (April 1, 2016), recurrence was present in one of the patients (8.3%) who did not receive a replacement pigtail stent (Figure 2). In four patients received replacement of SEMS with a 7-Fr plastic double pigtail stent, the stent was kept permanently in all of those patients.

**Table 1:** Patient characteristics

**Characteristics**	
Age, mean ± SD, yr	76.3 ± 12.1
Sex, male/female	9/3
Underlying condition	
III	66.7% (8/12)
IV	33.3% (4/12)
Advanced malignancy	8.3% (1/12)
White blood cell count (mean, range)	14525 (9100-21300) per μL
C-reactive protein (mean, range)	15.7 (2.0-32.7) mg/dL

**Table 2:** Outcomes of endoscopic ultrasound-guided gallbladder drainage

Technical success rate	100% (12/12)
Functional success rate	100% (12/12)
Rate of removal	67% (8/12)
Rate of replacement	33% (4/12)
Adverse events	0% (0/12)
Recurrence of cholecystitis	8.3% (1/12)
Follow-up period, days [median, range]	304 (78-1492)
Patient status on follow-up	
Alive	91.7% (11/12)
Dead	8.3% (1/12)

SEMS: Self-expandable metal stent.

**Figure 3:**
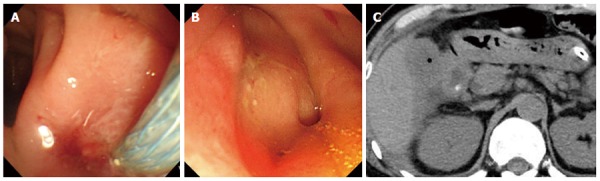
Esophagogastroduodenoscopy and computed tomography. A: Esophagogastroduodenoscopy image of deployment of the metal stent in the duodenum; B: Esophagogastroduodenoscopy image of the fistula 1 wk after removal of the metal stent; C: Computed tomography after removal of the metal stent showing air image in the gallbladder.

Before removal of the SEMS, gallstones did not remain in the gallbladder in all cases. One week after removal of the SEMS, air in the gallbladder was imaged by CT in nine cases (Figure 3). The other three cases that did not show air images in the gallbladder were cases in which the double pigtail plastic stents were not deployed. Fistulography was performed in eight cases that did not undergo replacement of the stent. Among these, fistulography images of the gallbladder were obtained in three cases. In total, the fistula was confirmed by CT and/or fistulography in 9 of 12 cases. Cystic duct patency was confirmed by fistulography and/or EUS before as well as 1 week after removal of the SEMS in all cases.

## DISCUSSION

The aim of the current study was to evaluate the feasibility of EUS-GBD for patients with acute calculous cholecystitis, who were deemed unsuitable for cholecystectomy. In this study, both technical and clinical success was achieved in the treatment of acute cholecystitis in all 12 patients. One of the risks of EUS-GBD is bile leakage into the peritoneal space, which can cause bile peritonitis. The bile leakage is caused by migration of the stent exposing the gap between the puncture tract and the stent[5,6,8]. In the present study, several techniques were used to avoid such bile leakage. Firstly, the guidewire was inserted until at least two full coils were in the lumen. The gallbladder lumen has more space for coiling than the bile duct, and yields better stability. Secondly, we irrigated the gallbladder lumen with saline solution after puncturing the gallbladder and before proceeding to the next step. This irrigation procedure may reduce the chance of peritonitis due to bile leakage during dilation. We also used SEMSs in our study, and, compared with plastic stents, SEMSs are better at sealing the gap between the stent and the needle tracts in the gallbladder wall, thus preventing bile leakage[8]. As a result, no adverse events occurred in this study. In a systematic review of EUS-guided biliary drainage by Wang et al[18] in 2016, the rate of adverse events was 38.46% in the group in which cystotomes were used during dilation of the puncture site, which was higher than that in the group in which dilators or balloons were used. Dilation of the puncture site was performed by biliary dilation and/or balloon catheters without using electrocautery in this study. This might be another reason why there were no adverse events in this study.

During a long-term follow-up with a median period of 275 d, Choi et al[19] reported that stent distal migration was noted in two patients (3.6%), one at 170 d and the other at 303 d post-EUS-GBD. They also reported recurrence of acute cholecystitis due to food impaction. To avoid stent migration and food impaction into the gallbladder, we either removed the SEMS, or replaced it with a pigtail plastic stent, 4 wk after EUS-GBD. In our study, there was neither stent migration nor food impaction. Performance of these additional procedures after EUS-GBD may prevent such complications.

Recently, the use of lumen-apposing metal stents (LAMS) with anchor flanges and flares for EUS-GBD resulted in excellent outcomes[13,15,20]. With a LAMS, the distance between the gastrointestinal tract and the gallbladder needs to be 1 cm or less[19]. In terms of this, a conventional biliary SEMS may have allowed us more freedom in selecting the puncture site, although this is not certain because the distance was 1 cm or less in all cases in this study.

In a study examining the use of LAMS for high-risk surgical patients with acutecholecystitis[20], Walter et al[20] reported that technical success was 90%, and clinical success was 96%, and that no migration was seen in any patients. In 15 of the 27 patients with technical success, LAMS were removed approximately 3 mo after EUS-GBD, whereas they were left in place in the other 12 patients. Removal of the LAMS was not achieved due to tissue overgrowth in two patients. Two patients also developed a LAMS obstruction. Thus, long-term deployment of metal stents in EUS-GBD could cause adverse events, including food impaction. Therefore, early removal of the metal stent after EUS-GBD, at a time of around 4 wk (as in the present study), may be considered desirable. However, we did observe a recurrence of acute cholecystitis in one patient (8.3%), where the SEMS was not replaced with a pigtail stent. There is a possibility that this patient recurred cholecystitis due to uncertain small gallstones or sludge remaining after EUS-GBD. Therefore, replacement with a pigtail plastic stent may be helpful for avoiding recurrence. Another reason for the low recurrence rate in this study might be that no gallstones remained in the gallbladder before removal of the SEMS in all cases.

Moon et al[21] reported that gross pathology showed adherence of the gallbladder to the stomach wall around the site of cholecystogastrostomy 4 wk after LAMS removal in an animal study. We also performed a preliminary examination of EUS-guided biliary drainage using a conventional biliary SEMS in an animal study using five pigs. We found that at autopsy 1 wk after the procedure, fistulas were created between the bile duct and duodenum in all pigs[22]. Thus, a strong fistula might develop between the gallbladder and the gastrointestinal tract within 4 wk using a conventional biliary SEMS as well as a LAMS. This study has a few limitations. Firstly, the number of EUS-GBD cases was low, and all cases were from a single institute. Secondly, the indications for EUS-GBD were limited to those patients deemed unsuitable for cholecystectomy. A larger study comparing the efficacy and safety of EUS-GBD with and without early SEMS removal is warranted. However, a large number of institutions are needed to obtain the required number of patients, otherwise the criteria used for patient selection should be less strict.

In a systematic review of EUS-GBD, LAMS seemed to have a high potential in terms of efficacy and safety; however, the technical success of LAMS (91.5%) was lower than that of conventional biliary SEMS (98.6%)[23]. Further studies including long-term results are required to investigate whether SEMS or LAMS are better for EUS-GBD.EUS-GBD with SEMS is a possible alternative treatment for acute cholecystitis. Long-term outcomes after removal of SEMS were promising. Removal of the SEMS after SEMS placement and improvement of symptoms might avoid migration of the stent and recurrence of cholecystitis due to food impaction.

## COMMENTS

### Background

Laparoscopic cholecystectomy is the standard treatment for acute cholecystitis caused by cholecystolithiasis. For patients at high surgical risk, percutaneous transhepatic gallbladder aspiration (PTGBA) or percutaneous transhepatic gallbladder drainage (PTGBD) can be selected for treatment of cholecystitis. However, the efficacy rate of PTGBA is insufficient (61%-77%), and PTGBD involves an external drainage tube, which decreases the ability of the patient to carry out their normal daily activities. Recently, endoscopic ultrasound-guided gallbladder drainage (EUS-GBD) was developed for acute cholecystitis.

### Research frontiers

There were few reports on long term outcomes of EUS-GBD. This study, the Long-term outcomes after removal of self-expandable metal stent (SEMS), was first report and the results of this study contribute to clarifying the potential of this procedure for acute cholecystitis.

### Innovations and breakthroughs

In this study, EUS-GBD using SEMS was a useful for removal of gallstones in the gallbladder. Gallstones disappeared after EUS-GBD in all cases. During long-term follow-up period after the removal of the SEMS, the recurrence of the cholecystitis was seen in only one patient (8.3%) and there were no complications.

### Applications

This study suggests that EUS-GBD using SEMS and removal of the SEMS 4 wk after the procedure are useful for patients with cholecystitis who were deemed unsuitable for cholecystectomy.

### Peer-review

This study described the use of EUS-GBD for the treatment of acute cholecystitis in patients deemed unsuitable for surgical procedures. Long-term outcomes after removal of SEMS were promising. Removal of the SEMS after SEMS placement and improvement of symptoms might avoid migration of the stent and recurrence of cholecystitis due to food impaction. A larger study comparing the efficacy and safety of EUS-GBD with and without early SEMS removal is warranted.
